# Comparison of the chemical health risk assessment of exposure to metal fumes for the furnace operator of a foundry industry using quantitative and semi-quantitative methods

**DOI:** 10.1016/j.heliyon.2023.e12913

**Published:** 2023-01-11

**Authors:** Sara Karimi Zeverdegani, Zahra Ordudari, Azim Karimi, Reza Esmaeili, Mohammad Kazem Khorvash

**Affiliations:** a Department of Occupational Health and Safety Engineering, School of Health, Isfahan University of Medical Sciences, Isfahan, Iran; b Student Research Committee, Department of Occupational Health and Safety Engineering, School of Health, Isfahan University of Medical Sciences, Isfahan, Iran; c Faculty of Health, Safety and Environmental Engineering, Najafabad Branch, Islamic Azad University, Iran

**Keywords:** Risk assessment, Metal fume, Foundry, SQCRA, US-EPA

## Abstract

Heavy metals have several adverse effects on the workers' bodies due to their accumulation in the vital organs. Besides that, the current study aimed to assess the health risk of exposure to metal fumes for furnace operators working in a foundry industry based on the three different methods. The current sectional descriptive-analytical research conducted on a foundry industry in Isfahan (Iran) in 2022. Three common methods currently available, including the Semi-Quantitative Risk Assessment Method (SQRCA) and two methods provided based on the US-EPA provided technique, were used in this study. At first, the extent of people's exposure to metal fumes of Fe, Ni, Cr, and Mn was measured. Then, the chemical risk assessment of exposure to these metals' fumes was done using the three methods, and their results were compared. The SPSS Ver.25 has been used for data analysis and comparison in the current study. Results indicated that the furnace operator's exposure to all four metals was above the allowed limit of occupational exposure. The chemical risk assessment results also showed that in the first method (US-EPA-based), the risk of exposure for all workers was acceptable, while in the second method (SQCRA), the risk level of a majority of workers was medium, and in the third method (US-EPA-based), the risk level of a majority of workers was not acceptable. Comparing the methods showed that average risk scores in the first and second methods were significant compared to the exposure to fumes with equivalent concentration (P_value_<0.05). The average score of carcinogenicity risk in method 3 was significant compared to the concentration of chromium and nickel (P-_value_ < 0.05), but it was not significant for iron and manganese and the non-carcinogenic risk of chromium and nickel. Chemical exposure risk level for the furnace operator was approximately moderate in all three methods. In terms of complexity and information required to implement the method, all three methods were almost the same, with the difference that the results of the first method cannot be generalized to other people who have the same job conditions because individual information such as a person's weight is used to calculate its score.

## 1 Introduction

Foundry is the technique of molding the metals and alloys by melting, pouring the melt into a container called a mold, and then cooling and freezing it according to the shape of the mold container. This method is the oldest known process to obtain a desired form of metal. The chemical pollutants in the foundry include Sulfur dioxide, carbon monoxide, iron-sulfur, nitrogen oxides, and toxic heavy metals such as manganese, cadmium, nickel, copper and chromium, molybdenum, etc. [1]. The heavy metals, in addition to adverse environmental effects, can cause several harms to the workers in case of entering the body and accumulating in the vital organs [2]. These metals can enter the body through inhalation in occupational spaces and the environment, water, and agricultural products [3]. They can accumulate inside the human body and lead to disruption of the enzyme synthesis pathway, stimulation of the production of oxygen free radicals, and inhibition of enzymes by the reaction between the metal and the sulfhydryl group in the body of exposed people [4]. These metals include two general groups, essential and non-essential. Essential metals include copper, zinc, and iron, which play a role in the metabolic and physiological activities of the body, but their amounts should not exceed the permissible limit; otherwise, they will cause damage to the human body and other living organisms. On the other hand, non-essential metals include metals such as cadmium and lead, which do not play a specific role in the body's metabolic activities, and their presence has adverse effects on the health of human health and other living organisms [5,6]. For example, chromium and its compounds cause digestive problems, spleen and liver size reduction, respiratory distress and irritation, and skin necrosis. The Hexavalent chromium (chromium (VI)) is known as a definite carcinogenic compound (group 1) [7]. Nickel is also a hematotoxic, immunotoxic, neurotoxic, genotoxic, reproductive, pulmonary, nephrotoxic, hepatotoxic, and carcinogenic agent [8]. Manganese is also an essential element in the body, and excessive exposure to manganese oxide compounds through breathing can cause lung problems [9]. Molybdenum is another heavy metal, and excessive exposure to it as an environmental pollutant produced through various industrial processes leads to reproductive toxicity, especially for men. In addition to these harmful effects on health, it also adversely affects the nervous system of exposed people [10,11]. Also, excessive exposure to an element such as iron causes gastrointestinal effects such as gastrointestinal bleeding, vomiting, and diarrhea in the short term and shock, hypotension, lethargy, tachycardia, liver necrosis, metabolic acidosis, and sometimes death in the long term [4]. Considering the increasing use of dangerous and toxic chemicals such as heavy metals in steel, mining, and casting industries [12] and also, the adverse effects of these substances on human health [13], it is necessary to carry out a health risk assessment of chemicals in these industries, which helps the organization in making decisions in choosing the type and method of application of control measures to eliminate or reduce the emission of such particles [14]. Chemical risk assessment describes health side effects caused by chemical substances and is carried out by various organizations worldwide [15]. Risk assessment of chemical substances can be done qualitatively using risk matrices and quantitatively using mathematical equations [16]. One of the recently used qualitative risk assessment methods is the Singapore chemical health risk assessment, also known as the SQRCA [17]. In a study by Mehrifar et al. (2020) using this technique to assess the health risk of gases emitted from welding, the study showed that O3 and NO2 gases and chromium fumes had high-risk levels. Also, it was concluded in this study that welders have a high risk of exposure to fumes and gases caused by welding, and it is necessary to implement appropriate control measures for these people [18]. Quantitative risk assessment is usually based on the integrated risk information system provided by the US Environmental Protection Agency. Based on this, several studies have introduced quantitative health risk assessment techniques, known as US-EPA-based methods [14,19]. In a study entitled “Quantitative Health Risk Assessment of Exposure to Automotive Casting Dust” conducted by Tong et al., in 2019 using mathematical equations based on US-EPA, 276 samples from 6 jobs were collected. Moreover, to calculate the health risk of exposure to dust, the value of DALY (Disability-Adjusted Life Year) was calculated for different compounds. The results of the mentioned study showed that the extent of risk in the jobs of smelting, unloading, finishing followed by pouring, sand preparation, molding, and product manufacturing for the risk probability of more than 6–10 is approximately 85%, 90%, 90%, 75%, 70%, and 45%, respectively. Also, the sensitivity analysis showed that the factors of average time, exposure duration, inhalation speed, and dust concentration (C) greatly contribute to the health risks caused by exposure to dust. In addition, this study stated that workers exposed to unloading and finishing jobs had the highest DALY with a value of 48.64 [20]. In the study by Kermani et al. (2021), which aimed to identify the possible sources of the health risk of heavy metals with a diameter of PM2.5 and assess them, a US-EPA-based method was used for quantitative risk assessment. In this study, the calculated health risk of metals was very significant, such that the maximum risk in children was related to cadmium (6.61), and in adults, it was related to manganese (0.82). Also, the highest amount of HQ in children and adults was related to Chromium. Finally, the ILCR values for cadmium in both groups of children (1.19 × 10^−4^) and adults (4.81 × 10^−4^) showed a high risk of developing cancer in humans [21]. Based on the investigations and the fact that no study has been conducted on the chemical risk assessment of the “furnace operator” job to assess the exposure to metal fumes, the current study was conducted to fill this research gap. The results of such studies can help managers fully understand the health risks caused by fumes, chemicals, and dust in different industries and provide a scientific basis for management and decision-making related to health damage assessment. Therefore, in this study, three common chemical risk assessment methods were used for the “furnace operator” job in the foundry industry based on following objectives:

• Determining the type and concentration of metal fumes in the breathing air of furnace operators.
• Determining the risk score of chemical exposure to metal fumes using US-EPA methods in the furnace operators.
• Determining the risk score of chemical exposure to metal fumes by SQRCA method in furnace operators.
• Comparison of exposure risk rating in three studied methods.

## 2 Method

### 2.1 Research design

The present study is a descriptive-analytical and cross-sectional study conducted in a foundry in Isfahan. This study studied 10 furnace operators (6 furnace operators in hall 1 and 4 in hall 2). The reason for choosing furnace operators to conduct the study was the high risk of exposure of these people to various types of metal fumes caused by heavy metals released from induction furnaces based on the preliminary survey conducted in this industry. Therefore, the present study evaluated the chemical risk of exposure to metal fumes and compared the results of three available chemical risk assessment methods.

### 2.2 Measurement of metal fumes available in the air

The current study measured metal fumes based on the NIOSH 7300 method [22]. According to this method, a cellulose ester membrane filter with a pore size of 0.8 μm and a diameter of 30 mm, a nylon cyclone, an individual sampling pump with a sampling flow rate of 2 L per minute, and a digital calibrator were used for sampling. The sampling filter was connected to the worker's collar in their respiratory area using a holder, and metal fumes were collected in the filters with the help of an individual sampling pump. According to this method, the sampling time was determined from 1 to 4 h so that the concentration of fumes on the filter does not exceed 2 mg. Also, one control sample was collected per every 10 samples for the accuracy of sampling. After the sampling was finished, the samples were sealed and transferred to the laboratory for sample analysis, and metal fume analysis was performed with an Inductively Coupled Plasma Mass Spectrometer (ICP-MS).

### 2.3 Metal fumes chemical health risk assessment

#### 2.3.1 Method 1 (US-EPA-based)

This risk assessment method was proposed by the United States Environmental Protection Agency (US-EPA) assess the health risks of chemicals on human health [23]. According to the following guidelines, the mentioned method was based on the US-EPA [24]. In this risk assessment method, in the first step, the exposure dose to heavy metals through breathing ${ADD}_{inh}$ is calculated according to the following equation:

(1) $${ADD}_{inh}=\frac{C\times InhR\times EF\times ED}{PEF\times BW\times AT}$$

C: Concentration of the measured heavy metal; InhR: Inhalation rate per m^3^/day; EF: Exposure frequency per day/year; ED: Period of exposure per year; PEF: Particle Emission Factor is equal to 1.36 × 10^9^ m^3^/kg; BW: Body Weight per kg; AT: The average time for non-carcinogenic risk equals AT = ED × 365 and for carcinogenic risk equals AT = 74.8 × 365.

In this method, to calculate the non-carcinogenic risk of the compounds, the (HQ) risk coefficient for each compound should be calculated according to the following equation:

(2) $$HQ=\frac{{ADD}_{inh}}{RfD}$$

RfD: Reference dose of each compound.

In the next step, for the carcinogenic and non-carcinogenic risks, the hazard index (HI) value should be calculated based on the sum of the hazard coefficients caused by different compounds.

(3) $$HI=\sum {HQ}_{i}$$

If the hazard index is more than one in the risk assessment, it indicates that the concentration of the pollutant is higher than the standard concentration and may cause concern in society. If this number is lower than one, it is expected that the exposed people will not get any disease over time [24].

In this method, to calculate the Carcinogenesis Risk (CR) during a person's lifetime, the following equation should be used:

(4) $$CR=\frac{{ADD}_{inh}}{SF}$$

SF: Severity Factor for each compound.

Finally, to calculate the total carcinogenic risk (TCR) caused by different compounds, the following equation was used:

(5) $$TCR=\sum {CR}_{i}$$

The World Health Organization has accepted the risk range of $10^{-5}$ and less and has determined values greater than $10^{-5}$ as the carcinogenic risk [25].

#### 2.3.2 Method 2 (Semi-Quantitative Risk Assessment Method)

The SQRA technique for the chemicals has been proposed by Singapore's institute for Workplace Safety and Health. This method systematically identifies risks related to chemicals, assesses exposure or the possibility of exposure, determines the level of risk, and prioritizes actions related to risk expression. The steps to perform this method follow the instructions below [17]. In this method, the two factors of Hazard Rate (HR) and Exposure Rate (ER) are important. The HR can be obtained by using the information related to the acute toxicity values of the substances and the LD_50_ and LC_50_ indices of the compounds, according to Table 1 [17].

**Table 1:** Acute toxicity degrees.

Risk Factor	LD50 absorbed orally in rats (mg/kg)	LD50 absorbed through the skin in rabbits or rats (mg/kg)	LC50 absorbed through the respiratory system in rats (mg/lit) for 4 h for gas and steam	LC50 absorbed through the respiratory system in rats (mg/lit) for airborne particles
2	2000<	2000<	20<	5 < LC50
3	200 < LD50 < 2000	400 < LD50 < 2000	2 < LC50 < 20	1 < LC50 < 5
4	25 < LD50 < 200	50 < LD50 < 400	0.5 < LD50 < 2	0.25 < LC50 < 1
5	<25	<50	<0.5	<0.25

In this method, for the calculation of the ER, first, the mean weekly weight of exposure should be calculated by the following equation, based on the real-time exposure available in air monitoring results.

(6) $$E=\frac{F\times D\times M}{W}$$

E: Weekly exposure (ppm or mg/m^3^); F: Frequency of weekly exposure (times per week); D: Average exposure time (h); M: Amount of exposure (ppm or mg/m^3^); W: Mean weekly working hours (54 h).

Then, the E factor calculated from the above equation is compared with the exposure limit values و, and the ER value is obtained in Table 2.

**Table 2:** ER calculation procedure.

Exposure rate (ER)	EOEL
1	<0.1
2	0.1–0.5
3	0.5–1
4	1–2
5	2≤

Finally, the risk score of exposure to each compound can be calculated using the following equation, and based on the calculated risk number; the risk rating can be obtained using Table 3:

(7) $$R=\sqrt{HR\times ER}$$

**Table 3:** Risk rank calculation procedure.

Risk score	Risk Ranking	Risk Rate	Necessary actions
0–1.7	Negligible	N	End of evaluation, reassessment every 5 years
1.7–2.8	Low	L	Non-continuous air sampling, reassessment every 5 year
2.8–3.5	Medium	M	Continuous air sampling, reassessment every 4 years
3.5–4.5	High	H	Completion of engineering controls, air sampling, personal protective equipment
4.5–5	Very high	VH	Completion of engineering controls, air sampling, personal protective equipment, setting emergency procedures

#### 2.3.3 Method 3 (US-EPA-based)

This chemical risk assessment method is also proposed based on the guidelines of the US EPA [26]. In this technique, Exposure Concentration (EC) and Exposure Time (ET) values were calculated according to working conditions, and inhalation absorption was considered as inhalation at 55% of exposure concentration (EC).

Therefore, the EC is calculated as follows:

(8) EC=CA×ET×EF×EDAT

EC: Exposure concentration in the air (μg/m^3^); CA: Concentration in the environment atmosphere; ET: Exposure time (h/d); EF: Exposure frequency (219 days/year); ED: Exposure duration (25 years); AT: The average time for non-carcinogenic risk is equal to AT = 25 y × 365 d/y × 24 h/d = 219,000 (h) and for carcinogenic risk equal to AT = 70 y x 365 d/y x 24 h/d = 631,200 (h).

In this method, to describe the non-carcinogenic risks of each compound, the hazard coefficient factor (HQ) is calculated using the following equation.

(9) $$HQ=\frac{EC}{RfD}$$

EC: Exposure concentration (μg/m^3^) in the 55% inhalation absorption.

If the HQ value is >1, there is a potential health risk from exposure, and if the HQ is <1, there is probably no acceptable risk of non-carcinogenic effects [26].

In this technique, to describe the carcinogenic risk of each compound during a person's lifetime, the Lifetime Carcinogenic Risk (LCR) is calculated as follows:

(10) LCR=IUR×EC

IUR: Inhalation unit risk (μg/m^3^).

In this technique also, based on the proposal of the World Health Organization, the risk range of $10^{-5}$ and less is acceptable and values greater than $10^{-5}$ are considered to be a carcinogenic risk [25]. All the risk factors used in the current study are provided in Table 4.

**Table 4:** Risk factors effective on calculations in the current study.

Risk factor	Description	Reference
Concentration	Concentration of pollutant in air	[27]
Exposure duration (ED)	Amount of time that an individual is exposed to the purpose pollutant (years)	[27]
Exposure time (ET)	Frequency that exposure occurs (h·day^−1^)	[27]
Exposure frequency (EF)	Frequency that exposure occurs (days·year^−1^)	[27]
Inhalation rate	Volume of air inhaled over a specified time	[27]
Body weight	Usually expressed in kilograms, used to normalize the dose	[27]
Slope factor	Measure of cancer risk from a lifetime exposure to an agent	[27]
Inhalation unit risk (IUR)	Estimation of the increased cancer risk from inhalation exposure to a concentration of 1 μg·m^−3^ for a lifetime	[27]
Inhalation reference concentration (RfC)	Estimation of a continuous inhalation exposure in the human population, which is likely to be without significant risk of adverse effects during a lifetime	[27]
Particle emission factor (PEF)	The constant value has been considered to be 1.36 × 10^9^ m^3^/kg	[24]
Average time (AT)	A constant value considered for non-carcinogenic risk equals AT = ED × 365 and AT = 74.8 × 365 for carcinogenic risk.	[24]
Weekly working hours (W)	The constant value has been considered to be 40 h	[17]

### 2.4 Data analysis

Data analysis was done using the SPSS Ver.25. The descriptive statistical tests have been used to evaluate the quantitative variables, mean, and standard deviation. The one-sample *t*-test has also been used to compare the final risk score obtained from the three workspace pollutants concentration assessment methods. The significance level in current research is less than 0.05 (P-value ≤ 0.05).

## 3 Results

### 3.1 Demographic data

The demographic data of the participants, including the height, weight, exposure time, frequency, duration, and concentration of particles in the air, can be seen in Table 5. The highest particle concentration in the air belongs to Fe, and the lowest belongs to Mo.

**Table 5:** Mean demographic data, exposure time and frequency, and weighted-temporal mean of the participants.

Parameter		Mean ± standard deviation
Height (cm)		173 ± 3.31
Weight (kg)		72.70 ± 8.73
Age (years old)		34 ± 3
Exposure duration (years)		9.10 ± 2.42
Exposure frequency (days·year^−1^)		312
exposure time (h·day^−1^)		9
(mg/m^3^) particles concentration in the air	Cr	0.00515
	Fe	0.28305
	Mn	0.0149
	Ni	0.0036

### 3.2 Health risk assessment results for different fumes

The RFD and SF values of metals to evaluate their carcinogenic and non-carcinogenic health risks are shown in Table 6.

**Table 6:** Reference doses (RfD) for noncarcinogens and slope factors (SF) for carcinogens.

Heavy metals	RFD_inh_ (mg/kg.d)	SF_inh_ (mg.d/kg)	Reference
Cr	3.00E−05	4.10E+01	[28]
Mn	1.43E−04	–	
Ni	2.06E−02	8.40E−02	
Fe	0.7	–	

The health risk assessment results of exposure to various released metals in the casting operations studied are presented in Tables 7–9. Table 7 shows the health risk assessment results of workers exposed to different metals using method 1. According to the results, using this method, the level of carcinogenic and non-carcinogenic risk of all workers exposed to all four evaluated metals was acceptable.

**Table 7:** Health risk evaluation of the different metals by the use of Method 1.

Worker	US-EPA 1 Health Risk Assessment											
	Cr				Fe (Non-Carcinogenicity)		Mn (Non-Carcinogenicity)		Ni			
	Non-Carcinogenicity		Carcinogenicity						Non-Carcinogenicity		Carcinogenicity	
	Risk Score	Risk Level	Risk Score	Risk Level	Risk Score	Risk Level	Risk Score	Risk Level	Risk Score	Risk Level	Risk Score	Risk Level
Worker 1	$1.92\times 10^{-9}$	A	$10^{-14}$	A	$2.2\times 10^{-11}$	A	$10^{-8}$	A	$1.26\times 10^{-9}$	A	$2.69\times 10^{-10}$	A
Worker 2	$1.36\times 10^{-9}$	A	$10^{-14}$	A	$5.5\times 10^{-11}$	A	$7\times 10^{-9}$	A	$1.13\times 10^{-9}$	A	$1.325\times 10^{-11}$	A
Worker 3	$2.13\times 10^{-8}$	A	$2\times 10^{-14}$	A	$4.9\times 10^{-10}$	A	$3.74\times 10^{-7}$	A	$2.29\times 10^{-11}$	A	$5.619\times 10^{-11}$	A
Worker 4	$1.02\times 10^{-8}$	A	$10^{-14}$	A	$9.4\times 10^{-10}$	A	$6.82\times 10^{-8}$	A	$1.07\times 10^{-11}$	A	$1.5991\times 10^{-10}$	A
Worker 5	$3.63\times 10^{-8}$	A	$3\times 10^{-14}$	A	$1.6\times 10^{-9}$	A	$2.28\times 10^{-7}$	A	$1.90\times 10^{-9}$	A	$2.6495\times 10^{-10}$	A
Worker 6	$2.26\times 10^{-8}$	A	$2\times 10^{-14}$	A	$1.1\times 10^{-9}$	A	$7.19\times 10^{-8}$	A	$3.91\times 10^{-10}$	A	$1.4024\times 10^{-10}$	A
Worker 7	$1.46\times 10^{-9}$	A	$10^{-14}$	A	$2.7\times 10^{-10}$	A	$6.24\times 10^{-7}$	A	$5.6\times 10^{2-10}$	A	$2.8304\times 10^{-10}$	A
Worker 8	$1.5\times 10^{-8}$	A	$10^{-14}$	A	$4.9\times 10^{-10}$	A	$1.68\times 10^{-7}$	A	$6.4\times 10^{-10}$	A	$9.792\times 10^{-11}$	A
Worker 9	$6.63\times 10^{-8}$	A	$5\times 10^{-14}$	A	$1.4\times 10^{-9}$	A	$3.30\times 10^{-7}$	A	$2.25\times 10^{-10}$	A	$4.7576\times 10^{-10}$	A
Worker 10	$3.74\times 10^{-8}$	A	$3\times 10^{-14}$	A	$1.5\times 10^{-9}$	A	$5.03\times 10^{-7}$	A	$1.06\times 10^{-9}$	A	$3.1498\times 10^{-10}$	A

A: Acceptable.

NA: Not Acceptable.

**Table 8:** Health risk evaluation of the different metals by the use of Method 2.

Worker	SQRCA Health Risk Assessment							
	Cr		Fe		Mn		Ni	
	Risk Score	Risk Level	Risk Score	Risk Level	Risk Score	Risk Level	Risk Score	Risk Level
Worker 1	2	2(L)	1.41	1(N)	2	2(L)	1.41	1(N)
Worker 2	2	2(L)	2	2(L)	2.45	2(L)	2	2(L)
Worker 3	3.16	3(M)	3.16	3(M)	3.16	3(M)	2.45	2(L)
Worker 4	2.45	2(L)	3.16	3(M)	2.83	3(M)	3.16	3(M)
Worker 5	3.16	3(M)	3.16	3(M)	3.16	3(M)	3.16	3(M)
Worker 6	2.83	3(M)	3.16	3(M)	2.83	3(M)	3.16	3(M)
Worker 7	2	2(L)	2.83	3(M)	3.16	3(M)	3.16	3(M)
Worker 8	2.83	3(M)	3.16	3(M)	3.16	3(M)	2.83	3(M)
Worker 9	3.16	3(M)	3.16	3(M)	3.16	3(M)	3.16	3(M)
Worker 10	3.16	3(M)	3.16	3(M)	3.16	3(M)	3.16	3(M)

N: Negligible risk ranking.

L: Low risk ranking.

M: Medium risk ranking.

**Table 9:** Health risk evaluation of the different metals by the use of Method 3.

Worker	US-EPA 2 Health Risk Assessment											
	Cr				Fe (Non-Carcinogenicity)		Mn (Non-Carcinogenicity)		Ni			
	Carcinogenicity		Non-Carcinogenicity						Non-Carcinogenicity		Carcinogenicity	
	Risk Score	Risk Level	Risk Score	Risk Level	Risk Score	Risk Level	Risk Score	Risk Level	Risk Score	Risk Level	Risk Score	Risk Level
Worker 1	$3.74\times 10^{-5}$	A	103.96	NA	0.24	A	29.25	NA	3.37	NA	$1.266\times 10^{-7}$	A
Worker 2	$3.76\times 10^{-5}$	A	103.96	NA	1.46	NA	19.35	NA	3.26	NA	$5.85\times 10^{-7}$	A
Worker 3	$7.48\times 10^{-4}$	A	2079.11	NA	8.91	NA	1125	NA	0.15	A	$2.7\times 10^{-6}$	A
Worker 4	$2.80\times 10^{-4}$	A	779.67	NA	13.37	NA	180	NA	0.031	A	$6.75\times 10^{-6}$	A
Worker 5	$9.35\times 10^{-4}$	A	2598.89	NA	22.28	NA	630	NA	5.18	NA	$1.17\times 10^{-5}$	A
Worker 6	$6.54\times 10^{-4}$	A	1819.22	NA	17.82	NA	270	NA	1.46	NA	$7.65\times 10^{-6}$	A
Worker 7	$5.61\times 10^{-5}$	A	155.93	NA	5.57	NA	1800	NA	1.91	NA	$1.305\times 10^{-5}$	A
Worker 8	$6.54\times 10^{-4}$	A	1819.22	NA	11.14	NA	630	NA	1.69	NA	$5.85\times 10^{-6}$	A
Worker 9	$1.87\times 10^{-3}$	A	5197.78	NA	16.71	NA	900	NA	0.67	A	$2.07\times 10^{-5}$	A
Worker 10	$9.35\times 10^{-4}$	A	2598.89	NA	20.05	NA	1350	NA	2.925	NA	$1.35\times 10^{-5}$	A

A: Acceptable.

NA: Not Acceptable.

Table 8 presents the health risk assessment results of 4 evaluated metals using method 2. According to the results of this method, Mn and Fe had a higher risk level than the other two metals. However, most of the workers had a medium level of exposure to the studied metals.

In Table 9, the results of the health risk assessment of the studied metals using method 3 can be seen. The results of this method showed that the carcinogenic risk level of Cr and Ni was acceptable among all workers. Meanwhile, the non-carcinogenic risk in most workers exposed to the studied metals was not acceptable.

### 3.3 Measurement of fumes concentration, comparison with the standard occupational exposure, determination of the risk score, and comparison of the three studied methods

The equivalent concentration of exposure and the occupational exposure limit based on Iran's standard, the score and level of exposure risk, and the comparison of the three studied methods are shown in Table 10. Furnace operators under study were exposed to chromium, iron, manganese, and nickel fumes, of which the lowest concentration equivalent to exposure belonged to chromium, and the highest belonged to iron. Among the measured fumes, the concentration of all studied fumes was evaluated to be above the permissible limit. As can be seen in Table 10, the four fumes under study were evaluated and ranked using three methods of the US-EPA, SQRCA, and mathematical equations. On the other hand, the risk assessment of all metals by method 1 was found to be acceptable. It should be noted that the risk rating of method 2 for most fumes was obtained one, two, and three, which indicates very low, low, and moderate exposure, respectively, and sufficient control with regular, irregular, and no maintenance.

**Table 10:** Equivalent concentration of exposure to the permissible exposure limit and comparison of the score of three risk assessment methods in the studied fumes.

Type of metal fume			Cr	Mn	Fe	Ni
C_TWA_			0668.0	0.3082	10.55	0.1836
Occupational exposure limit			0.05	0.1	10	0.1
Method 1	Carcinogen	Risk Score	$10^{-10}$	–	–	$10^{-4}$
		Risk Level	A	–	–	NA
	Non carcinogen	Risk Score	$10^{-4}$	$10^{-4}$	$10^{-4}$	$10^{-4}$
		Risk Level	A	A	A	A
P value			0.001<	<0.001	<0.001	<0.001
Method 2	Risk Score		2.67	2.9	2.83	2.67
	Risk Level		2(L)	3(M)	3(M)	2(L)
P value			0.001<	0.001	0.001<	0.001<
Method 3	Carcinogen	Risk Score	$6\times 10^{-4}$	–	–	0.043
		Risk Level	A	–	–	NA
	P value		0.001<	–	–	0.001
	Non carcinogen	Risk Score	1725.66	693.36	11.75	2.06
		Risk Level	NA	NA	NA	NA
P value			0.007	0.005	0.631	0.005

Significance level was considered as p-value < 0.05.

A: Acceptable.

NA: Not Acceptable.

In the risk assessment of the studied metals by Method 3, the carcinogenicity of chromium, nickel, and iron risk assessment was acceptable for a worker. Also, the non-carcinogenic risk assessment of chromium and manganese was acceptable for all workers and for most workers in terms of the nickel and iron's non-carcinogenic risk. In the following, the score of the three introduced risk assessment methods was compared with the equivalent exposure concentration in the studied fumes. The one-sample *t*-test showed that the average risk scores in method 1 and method 2 significantly differed from the equivalent exposure concentration of the fumes under study (P-value < 0.05). On the other hand, the mean score of carcinogenic risk in method 3 was significantly different (P-value < 0.05) from the equivalent concentration of exposure to chromium and nickel fumes; however, this difference was not significant for iron and manganese, as well as the non-carcinogenic risk of chromium and nickel.

## 4 Discussion

The present study was conducted in a foundry industry to assess the risk of chemical exposure of furnace operators to metal fumes and compare the results of chemical risk assessment using three methods of US-EPA, SQRCA, and mathematical equations. Casting is one of the essential parts of any construction industry, which is often done on a small scale with a small number of workers. The workers of the foundry industry are more exposed to harmful factors such as inhalation of metal fumes compared to the workers of other sectors in the traditional way [29]. Smelting and casting in traditional foundry industries, which is done with direct human intervention, is one of the most dangerous activities in the foundry industry [30]. Because furnace operators in the studied industry did smelting and casting, the present study assessed the chemical risk of furnace operators. This study has two general objectives. The first objective was to estimate the risk of chemical exposure of furnace operators to metal fumes. For this purpose, the intended metals were first measured according to the NIOSH 7300 method, and the study results showed that the exposure of furnace operators to hexavalent chromium, iron, manganese, and nickel was higher than the permissible exposure limit in the Iranian standard. A study conducted in India showed that workers in small and medium-scale foundry industries are more exposed to respiratory pollutants (above the standard limit) [31]. In another study conducted in Indonesia in 2020, it was reported that respiratory exposure to air pollutants in foundry workers was higher than the permissible limit [32]. The results of the mentioned studies are in line with the present study. However, no study has been conducted in Iran to evaluate the exposure of foundry workers to chemical pollutants, and the results of this study are the first results of the evaluation of exposure to metal fumes in the foundry industry. In the present study, the concentration of chromium, iron, manganese and nickel metals are 0.00515, 0.28305, 0.0149 and 0.0036 mg/m^3^ respectively and their risk levels in SQRA method are 2, 1, 2 and 1 respectively. It was estimated. In the study of Golbabai et al., in 2012, the concentration of nickel and chromium in welders was found to be 0.08252 and 0.01954 mg/m3, respectively [33]. In another study conducted by Shokrolahi et al., in 2020, the concentration of manganese and iron in a welding process was 0.1239667 and 0.527 μg/L, respectively [34]. In 2012, Hasani et al. reported the concentration of manganese in welders as 0.011 mg/m^3^ [35]. Also, in Mehrifar et al.'s study in 2020, the concentration of chromium, nickel, and manganese metals in a welding process was 3.19, 1.24, and 2.30 mg per cubic meter, respectively, and the risk level by SQRA method was 33, respectively. 2.1, 1.73 and 2.0 were estimated, which is almost consistent with the current study, even though the type of job was different [18]. On the other hand, the concentration obtained from metal fumes in different studies is sometimes significantly different from the present study, which can be attributed to the different types of jobs and environmental conditions. It should be noted that no study was found that measured the concentration of heavy metals in the furnaces of the casting process.

The level of exposure risk of furnace operators to the studied metal fumes (iron, manganese, nickel, and hexavalent chromium) was calculated to evaluate the chemical risk using the three mentioned methods. Table 7 showed that the calculated risk was acceptable for all 10 people, while the concentration of all 4 investigated metals was reported to be higher than the permissible exposure limit. Therefore, it is logical to calculate the exposure risk as not acceptable to confirm the method's effectiveness. The reason for this difference can be seen in the fact that the exposure concentration considers the risk level of people for 30 years of working. Still, in this study, the average working experience of people was about 9 years and considering that the exposure period is less than a third of the exposure period for the permissible level of exposure, it seems that the results obtained are logical. Besides these, the estimation of the exposure concentration in the work environment is through the measurement of the evaluated substance, the type of process and ventilation, but in some chemical substance risk assessment methods, including the first method used in this study (US-EPA), the exposure level of workers are also a function of their activity level (Inhalation rate) [23]. On the other hand, Table 9 shows that the exposure concentration and the obtained risk score are significantly and directly correlated, i.e., with the increase in the concentration, the value of the risk score has also increased, according to the calculation formula (direct relationship between the risk score and the concentration) of the risk score, this result is also logical. The results of chemical risk assessment using the first method show that the concentration of chemicals in the workplace alone is not enough to determine the risk level of chemical exposure, and other parameters are effective in determining the risk level. However, the strong and significant relationship between the concentration of chemicals and this method's final score shows that the concentration's role in the workplace greatly impacts determining the risk level of chemical exposure.

In risk assessment using the second method, based on the results shown in Table 8, the workers' scores and level of risk assessment varied from insignificant risk level to medium risk level, but the highest level of risk obtained was reported as the medium. On the other hand, the correlation between the risk score in this method and the concentration of pollutants in the work environment has been direct and significant, i.e., with the increase in the concentration of substances, the risk score has also increased significantly. Nevertheless, in this method, carcinogenic and non-carcinogenic risks of substances are not calculated separately. In the chemical risk assessment using the second method, although the exposure concentration of the work environment is higher than the permissible exposure concentration, the scores and the medium risk level have been obtained, which is indicative of the tiny effects of concentration in determining the final score of this method and ultimately determining the risk level using this method.

In the third risk assessment method, the carcinogenic risk was acceptable for all the studied workers. The non-carcinogenic risk was reported to be acceptable for chromium, manganese, and iron, except for one worker. However, the non-carcinogenic risk for nickel was found to be not acceptable in most cases. The average risk score in this method was direct and significant with Cr and Ni concentration but not significant in other cases.

The first and second methods had a better correlation than the correlation between the measured concentration with the final score and the risk level. However, to better judge which of the three mentioned methods is more suitable for evaluating the chemical risk of furnace operators? Apart from the correlation between the final score of the results of each of the methods with the measured concentration, other parameters such as the complexity of the method, the time-consuming nature of the work method, easy access to the necessary information to perform the procedure, an easier judgment of the results, and prioritization of risks were investigated.

The comparison of the methods used in the current study showed that the second method offers better stratification than the other two methods, and it is easier to judge the level of risk than the other two methods because the second method categorizes the level of risk into four levels. In comparison, the other two methods categorize the level of risk in only two levels (acceptable or not acceptable). In terms of complexity and information required to implement the method, all three methods were almost the same, with the difference that the results of the first method cannot be generalized to other people who have the same job conditions because individual information such as a person's weight is used to calculate its score. In comparison, the results of the other two methods can be generalized to other people who have the same job. Therefore, using the first method, where the objective is to evaluate occupations, and it is necessary to evaluate people working in a job title, is considered a weakness for this method. There is no significant difference between the three mentioned methods regarding the time required and the cost needed to perform the risk assessment. Therefore, by summarizing all the above parameters, it seems that the use of each method depends a lot on the purpose of risk assessment. For example, when there is a need for occupational risk assessment and the carcinogenesis risk must also be checked, among the three mentioned methods, the third method will provide better results and is more suitable. Alternatively, if an individual risk assessment is intended and the carcinogenic risk is also considered, the first method is a more suitable option, and also, when there is a need for more precise prioritization, the second method is more suitable. In total, the final results of all three methods are almost similar. Kerami Mosafer et al. (2022) aimed to assess the risk of exposure to formaldehyde in histopathology laboratory operators using three US-EPA-based methods based on the US-EPA. In this study, it is reported that the results of the investigated methods to determine the carcinogenic risk of formaldehyde were almost similar, which is in line with the results of the current study [36]. In the research by Kalate et al. (2020), a US-EPA-based method (similar to the third method of the current study) was used to assess the risk of chemical exposure to heavy metals among the maintenance and laboratory personnel of a mineral salts manufacturing company. In the mentioned research, despite the exposure of workers to metal fumes was less than the recommended permissible limits, but the carcinogenic and non-carcinogenic risks of the substances were not negligible, so the authors are concerned about the using ventilation systems in the workplace and also implementation more control measures were recommended [37]. These results were consistent with the results of the present study, which makes it necessary to implement control measures such as reducing the working hours of operators and using ventilation systems to reduce the risk ranking of exposed workers. A 2020 study aimed at assessing the risk of exposure to heavy metals from ambient (street) dust used US-EPA-based methods for heavy metal health risk assessment [38]. Carcinogenic and non-carcinogenic risk of heavy metals in the participants of the mentioned study was within the tolerable risk range. However, the carcinogenic and non-carcinogenic risk obtained in the present study was not within the tolerable range recommended by the method. Considering that the present study was conducted in an industrial environment, the amount of exposure of employees and the hours of exposure of workers were higher compared to the aforementioned study, which led to a higher exposure risk of employees.

The advantage of the present study is the use of three different methods to assess the health risk of different metal fumes in furnace operators in a foundry industry. Comparing these three methods will help the reader to choose the best method for health risk assessment of metal fumes. One of the limitations of the current study was the low number of participants due to the small available population working in the studied industry. Therefore, it is suggested to select more samples from several different foundry industries in future studies. Another limitation of this study was the lack of proper participation of personnel in the sampling process. However, with the help of the management of the organization, sampling was done with high accuracy.

## 5 Conclusion

Furnace operators in the foundry industry are facing the impermissible concentration of heavy metal fumes, which need to be prioritized in the intervention programs of technical and engineering controls. In terms of risk classification also, the risk level of chemical exposure for furnace operators was approximately obtained using all three evaluation methods, which indicates the existence of the risk of exposure to metal fumes for furnace operators, which has necessitated the implementation of interventions to control pollutants in the work environment. Each of the studied methods in this research can be used with consideration for the parameters such as the complexity of the method, easy access to the information required to perform the method, the time-consuming nature of the method, easy judgment about the results, and risk classification for managing the outcomes and prioritizing risks depending on the purpose of risk assessment. However, the comparison of the results of the three methods showed that method one is more accurate in calculating the risk ranking of heavy metal due to the fact that each person's personal information (such as body weight) is needed to health risk assessment.

## Ethics approval

This study was approved as a research project in the ethics committee of Isfahan University of Medical Sciences with code number IR. MUI.RESEARCH.REC.1400.495.

## Author contribution statement

Sara Karimi Zeverdegani: Conceived and designed the experiments.

Zahra Ordudari: Analyzed and interpreted the data; Wrote the paper.

Azim Karimi: Performed the experiments.

Reza Esmaeili: Contributed reagents, materials, analysis tools or data; Wrote the paper.

Mohammad Kazem Khorvash: Contributed reagents, materials, analysis tools or data.

## Funding statement

This research did not receive any specific grant from funding agencies in the public, commercial, or not-for-profit sectors.

## Data availability statement

Data will be made available on request.

## Declaration of interest's statement

The authors declare no competing interests.
